# Supplementary material for: Design and Synthesis of New Anthranyl Phenylhydrazides: Antileishmanial Activity and Structure–Activity Relationship
Source: Pharmaceuticals (Basel). 2023 Aug 9;16(8):1120. doi: 10.3390/ph16081120 (PMC10458276; doi:10.3390/ph16081120)
Supplement: Supplementary file 1 [file pharmaceuticals-16-01120-s001.zip › pharmaceuticals-2506172-supplementary.pdf]

Article

Article

Design and Synthesis of New Anthranyl Phenylhydrazides: Antileishmanial Activity and Structure–Activity Relationship

Claudia do Carmo Maquiaveli [1,*], Edson Roberto da Silva [1,*], Barbara Hild de Jesus [1], Caio Eduardo Oliveira Monteiro [1], Tiago Rodrigues Navarro [2], Luiz Octavio Pereira Branco [2], Isabela Souza dos Santos [2], Nanashara Figueiredo Reis [2], Arieli Bernardo Portugal [3,4], João Luiz Mendes Wanderley [3], André Borges Farias [5,6], Nelilma Correia Romeiro [6] and Evanoel Crizanto de Lima [2,*]

1 Laboratório de Farmacologia e Bioquímica (LFBq), Departamento de Medicina Veterinária, Universidade de São Paulo Faculdade de Zootecnia e Engenharia de Alimentos, Av. Duque de Caxias Norte 225, Pirassununga 13635-900, SP, Brazil
2 Laboratório de Catálise e Síntese de Substâncias Bioativas, Instituto Multidisciplinar de Química, CM UFRJ-Macaé, Universidade Federal do Rio de Janeiro, Macaé CEP 27971-525, RJ, Brazil
3 Laboratório de Imunoparasitologia, Instituto de Ciências Médicas, Centro Multidisciplinar UFRJ, Macaé CEP 27979-000, RJ, Brazil
4 Programa de Pós Graduação em Biociências e Biotecnologia, Universidade Estadual do Norte Fluminense, Campos dos Goytacazes CEP 28013-602, RJ, Brazil
5 Unidad Académica de Yucatán, Instituto de Investigaciones en Matemáticas Aplicadas y en Sistemas, Universidad Nacional Autónoma de México, Mérida 97302, Yucatán, Mexico
6 Integrated Laboratory of Scientific Computing (LICC), Federal University of Rio de Janeiro (UFRJ)—Campus Macaé, Aluízio Silva Gomes Avenue 50, Granjas Cavaleiros, Macaé 27930-560, RJ, Brazil
* Correspondence: cmaquiaveli40@gmail.com (C.d.C.M.); edsilva@usp.br (E.R.d.S.); evanoel.crizanto@gmail.com (E.C.d.L.)

Supporting Information

Table S1. Targets predicted by chemogenomics.

Uniprot	Target name	Organism
Q01782	Pteridine reductase 1	Leishmania major
Q27686	Pyruvate kinase	Leishmania mexicana
O96394	Arginase	Leishmania amazonensis

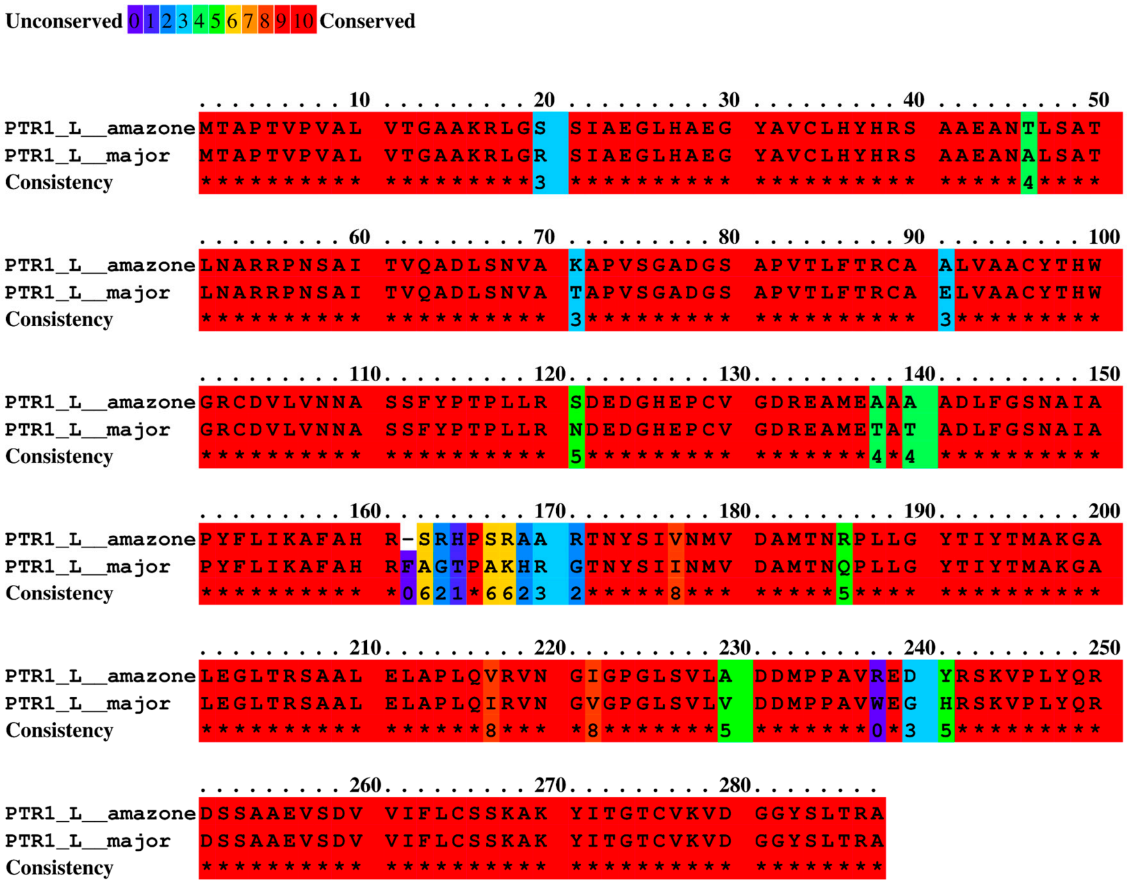

Figure S1. Alignment between PTR1 of *L.amazonensis* and *L. major*.

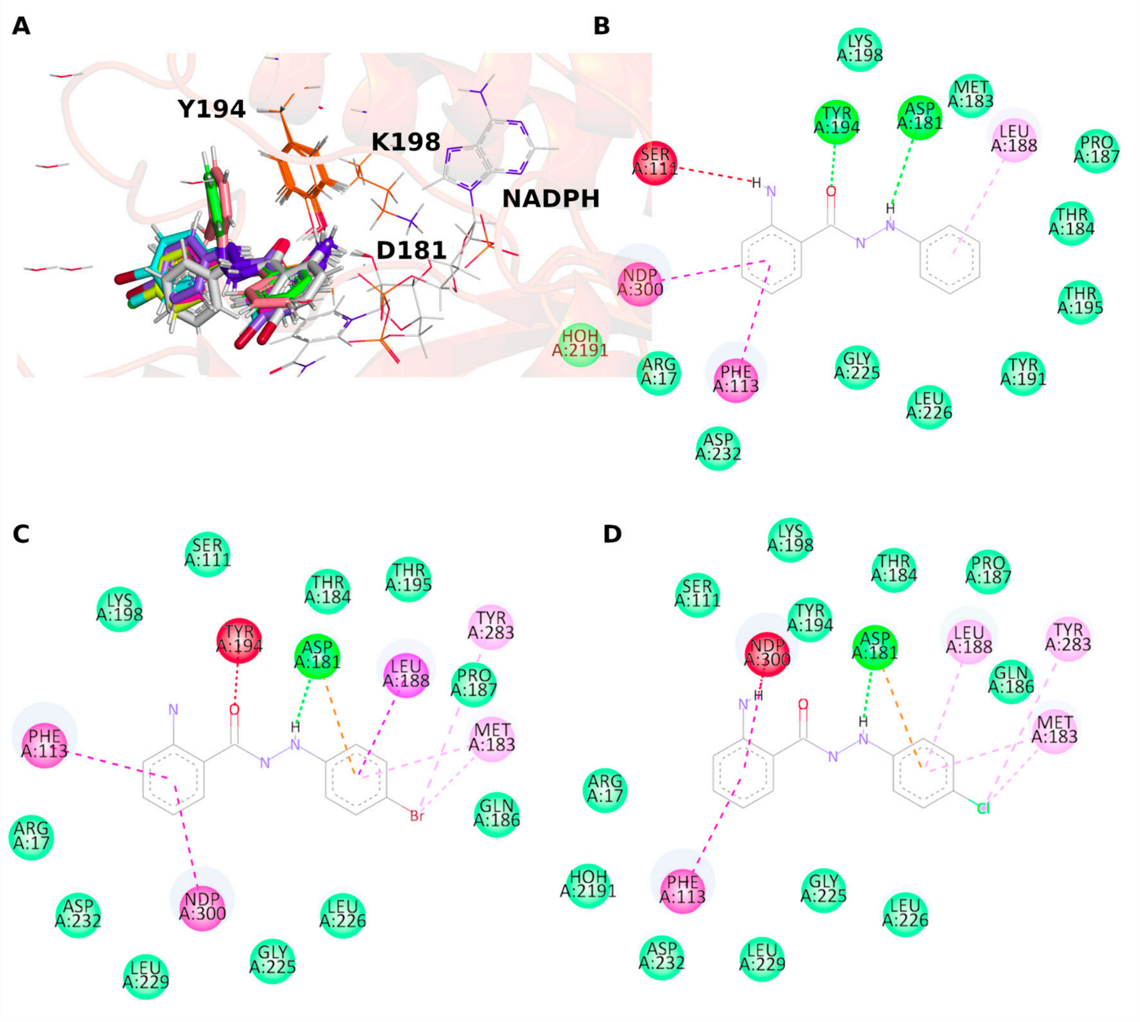

Figure S2. (**A**) Overlay of Putative binding pose. Docking analysis of inhibitors (**B**) 1a (**C**) 1d and (**D**) 1e. Interactions are colored by type: hydrogen bond, van der Waals, alkyl, Pi-stacked, halogen, unfavorable donor-donor, and Pi-anion/cation are shown by colors green, light green, light pink, pink, cyan, red, and gold, respectively.

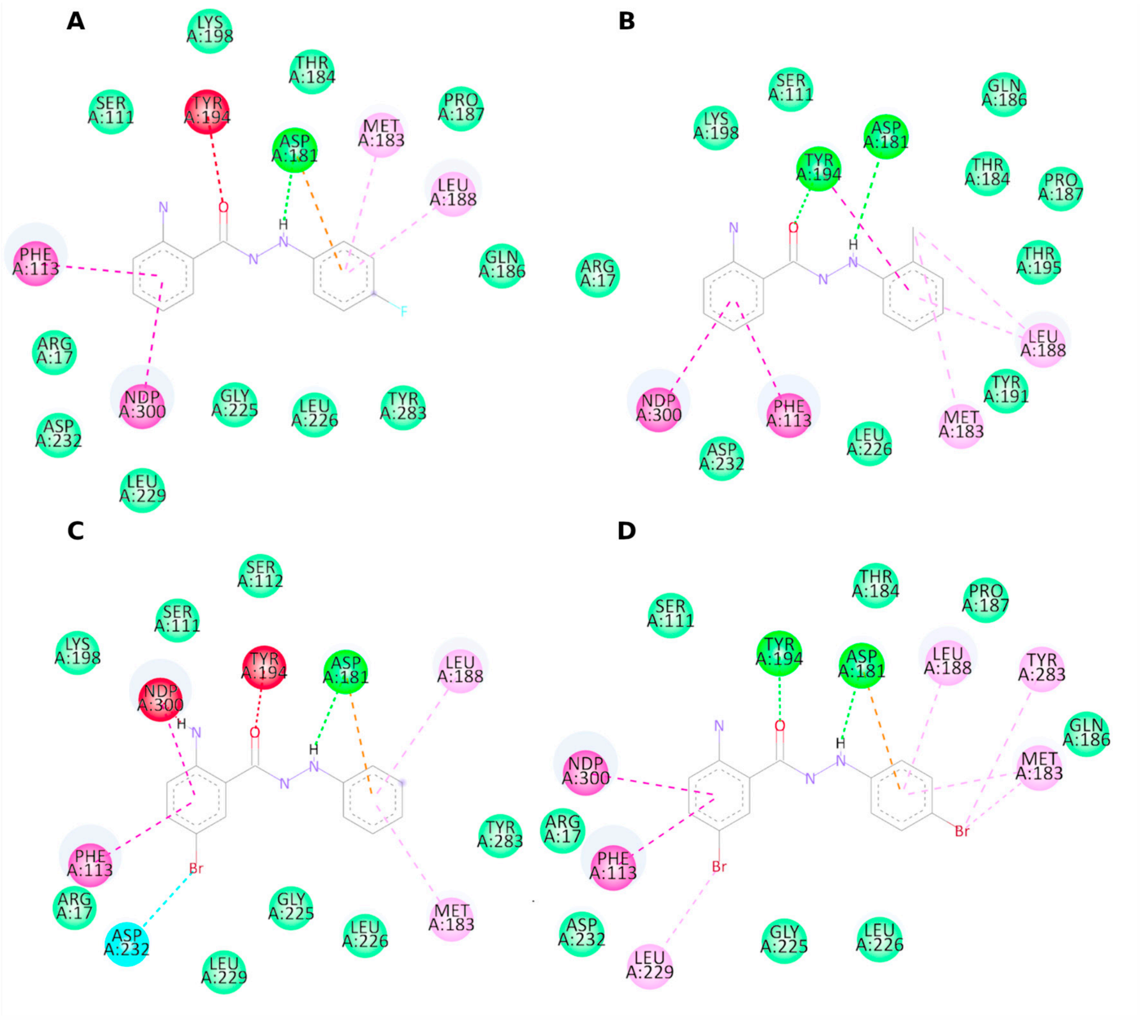

Figure S3.(**A**) Docking analysis of inhibitors 1f (**B**) 1j (**C**) 1c and (**D**) 1g. Interactions are colored by type: hydrogen bond, van der Waals, alkyl, Pi-stacked, halogen, unfavorable donor-donor, and Pi-anion/cation are shown by colors green, light green, light pink, pink, cyan, red, and gold, respectively.

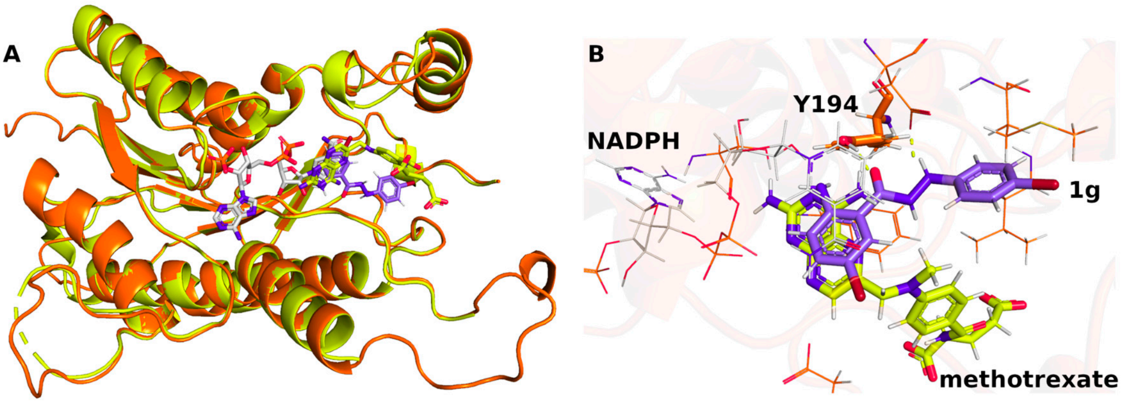

Figure S4. (**A**) Structural alignment between PTR1 from *L. major* (yellow) and *L. amazonensis* (orange). (**B**) Comparison between docking pose of inhibitor 1g and methotrexate co-crystallized in PTR1

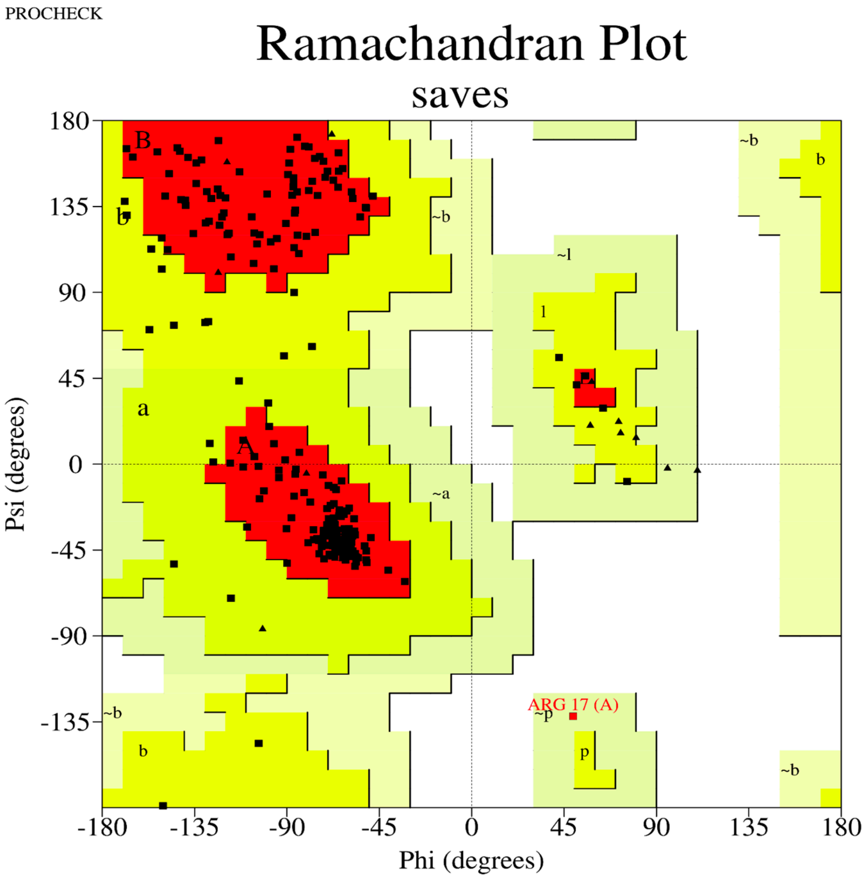

Plot statistics

Residues in most favoured regions [A,B,L]	205	89.5%
Residues in additional allowed regions [a,b,l,p]	23	10.0%
Residues in generously allowed regions [~a,~b,~l,~p]	1	0.4%
Residues in disallowed regions	0	0.0%
	----	------
Number of non-glycine and non-proline residues	229	100.0%
Number of end-residues (excl. Gly and Pro)	8	
Number of glycine residues (shown as triangles)	18	
Number of proline residues	14	

Total number of residues	269	

Figure S5. Ramachandran plot of PTR1 model from *L. amazonensis* obtained in AlphaFold.

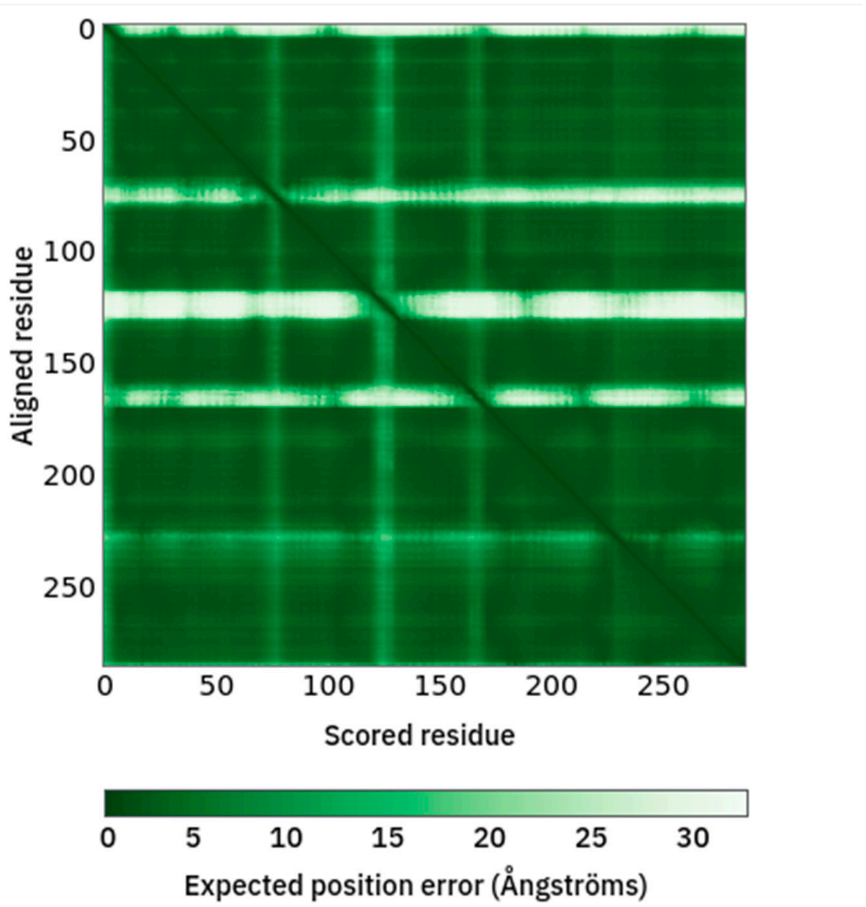

Figure S6. Predicted error of PTR1 model from *L. amazonensis* obtained in AlphaFold.

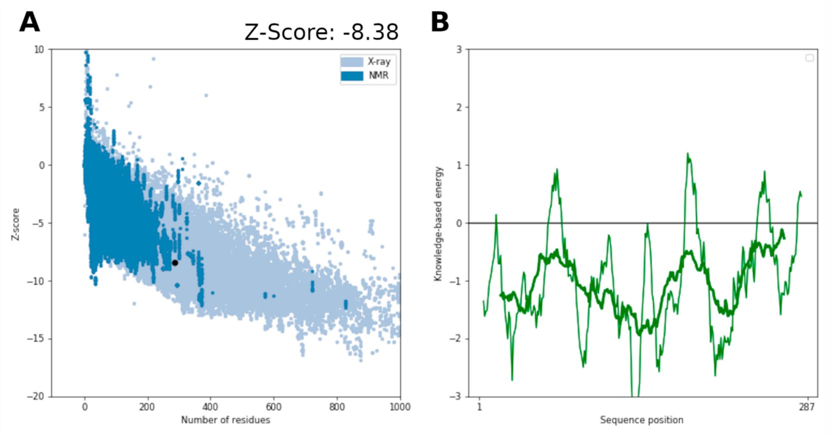

Figure S7. (A) Comparison of z-score of our model against all protein chains in PDB determined by X-ray crystallography (light blue) or NMR spectroscopy (dark blue). (B) Local model quality observed by residue energies averaged in two windows size 10 (light green) and 40 (green).
